# Exploring the relationship between SARS‐CoV‐2 variants, illness severity at presentation, in‐hospital mortality and COVID‐19 vaccination in a low middle‐income country: A retrospective cross‐sectional study

**DOI:** 10.1002/hsr2.1703

**Published:** 2023-12-01

**Authors:** Muhammad Zain Mushtaq, Nosheen Nasir, Syed Faisal Mahmood, Sara Khan, Akbar Kanji, Asghar Nasir, M. Asif Syed, Uzma Bashir Aamir, Zahra Hasan

**Affiliations:** ^1^ Department of Medicine The Aga Khan University Karachi Pakistan; ^2^ Department of Pathology and Laboratory Medicine The Aga Khan University Karachi Pakistan; ^3^ Department of Health Government of Sindh Hyderabad Pakistan; ^4^ Country Office World Health Organization Islamabad Pakistan

**Keywords:** COVID‐19, Omicron, Pakistan, severity, vaccinations

## Abstract

**Background and Aims:**

COVID‐19 morbidity and mortality varied globally through the pandemic*.* We studied the relationship of SARS‐CoV‐2 variants of concern (VOC) with COVID‐19 severity and mortality among hospitalized patients in Pakistan.

**Methods:**

A retrospective review of clinical, laboratory, and vaccination data of 197 COVID‐19 adult patients at the Aga Khan University Hospital, Karachi between April 2021, and February 2022 was performed. SARS‐CoV‐2 VOC identified in respiratory samples were analyzed. Univariate and multivariate analysis was conducted to identify factors associated with COVID‐19 outcomes.

**Results:**

The median age of cases was 55 years and 51.8% were males. Twenty‐four percent of females were pregnant. Of COVID‐19 cases, 48.2% had nonsevere disease, while 52.8% had severe/critical disease. Hypertension (48%) and diabetes mellitus (41%) were common comorbids. SARS‐CoV‐2 VOC identified comprised; Omicron (55.3%), Beta (14.7%), Alpha (13.7%), Delta (12.7%), and Gamma (3.6%) variants. Most (59.7%) study subjects were unvaccinated. Of vaccines, 88% had received inactivated virus COVID‐19 vaccines. Increased risk of severe disease was associated with age ≥50 years (odds ratio [OR]: 5.73; 95% confidence interval [CI]: [2.45–13.7]), as well as with diabetes mellitus (OR: 4.24; 95% CI: [1.82–9.85]). Full vaccination (OR: 0.25; 95% CI: [0.11–0.58]) or infection with Omicron (OR: 0.42; 95% CI: [0.23–0.74]) was associated with reduced disease severity. The risk of mortality increased with age ≥50 years (OR: 5.07; 95% CI: [1.92–13.42]) and a history of myocardial infarction (OR: 5.11; 95% CI: [1.45–17.93]) whilst, infection with Omicron was found to reduce the risk (OR: 0.22; 95% CI: [0.10–0.53]).

**Conclusion:**

Our study describes the relationship between the severity of COVID‐19, in‐hospital mortality in relation to SARS‐CoV‐2 variants, and the impact of COVID‐19 vaccination in Pakistan. Outcomes were more favorable in younger individuals, after vaccinations and with Omicron variant infections. Most cases received inactivated virus vaccines therefore these data highlight the protection provided against severe COVID‐19.

## 1 INTRODUCTION

COVID‐19 (coronavirus disease 2019) was been diagnosed in greater than 690 million cases with a global death toll of 6.89 million as of 10th March 2023.^1^ Global epidemiology of COVID‐19 varied greatly, with the highest burden of deaths to date observed in the United States, Brazil, and India.^1^ Low middle‐income countries such as Pakistan and those in Sub‐Saharan and East Africa were relatively spared and mortality due to COVID‐19 was lower.^2^, ^3^

COVID‐19 is an illness marked by respiratory difficulties, high body temperatures, coughing, tiredness, pneumonia, and muscular discomfort.^4^ COVID‐19 cases can be categorized into nonsevere with mild symptoms, severe cases that require oxygen support, and critical disease that require either noninvasive or invasive mechanical ventilation.^5^ The prevalence of various persistent symptoms among COVID‐19 survivors, including psychological symptoms such as depression, as well as physical symptoms like chest pain, dizziness, hair loss, weight loss, palpitations, and sleep difficulties.^6^ Fajar et al.^7^ reported persistent fatigue, anosmia, headaches, myalgia, and joint pain among a significant proportion of COVID‐19 survivors.

Pakistan experienced five pandemic waves between 2020 and 2022, between March and July 2020, October 2020 and January 2021, March and May 2021, July and September 2021 and between December and February 2022.^8^ As of June 16, 2023, COVID‐19 was diagnosed in 1.58 million cases in Pakistan with about 31,000 related deaths.^1^ Of the COVID‐19 cases in Pakistan, 36% cases were from Sindh province with 40% of those reported from the city of Karachi.^9^ The case fatality rate (CFR) has been 2% with some regional variations. It is of interest to understand the factors associated with COVID‐19 severity in the local context.

The Aga Khan University Hospital (AKUH), Karachi, Pakistan was at the forefront of treating COVID‐19 patients in the early pandemic period. In a study from our center during the early pandemic period in 2020, it was seen that 30% of patients had severe to critical disease at presentation with age and critical disease (identified by septic shock and multiorgan dysfunction) were independently associated with mortality.^10^

Within a few months of the start of the pandemic in December 2019, it was seen that SARS‐CoV‐2 acquired mutations particularly in its spike glycoprotein, which was associated with entry to host cells. Variants carrying mutations in the spike gene were described by the World Health Organization (WHO) to be variants of concern (VOC) and include, Alpha (B.1.1.7), Beta (B.1.351), Gamma (P.1), Delta (B.1.617.2), and Omicron (B.1.1.529).^11^ VOC exhibit genetic modifications linked to increased transmissibility, more severe disease, reduced efficacy of vaccinations or medical treatment.^12^ The Omicron variant which emerged in the latter half of 2021, had more than 50 mutations across the genome, resulting in reduced vaccine efficacy with a greater chance of breakthrough infections.^13^ Omicron was found to be more transmissible compared with other variants, with a greater chance of re‐infections. However, it was associated with decreased in‐hospital mortality rates.^13^

G, L, and S clade SARS‐CoV‐2 strains were shown to be present in the first wave of COVID‐19 in Pakistan between March and August 2020.^14^ Alpha variants were identified in January 2021 and there was a shift in the predominance of VOC from Alpha to Delta variants between April and July 2021, and then from Delta to Omicron variants between December and February 2022.^15^, ^16^, ^17^ The Omicron variant wave reached its peak in Pakistan in January 2022, resulting in the highest number of cases since the beginning of the pandemic.^1^, ^16^

COVID‐19 vaccinations were first introduced at the start of 2021^18^ and rolled out in stages based on both access and availability in different populations. Vaccinations slowed slowed down the pandemic, controlling both morbidity and mortality from COVID‐19.^19^ In Pakistan, inactivated type of vaccines were introduced in February 2021 first in healthcare workers and subsequently staged in older age groups and other populations. Most of the vaccines administered in 2021 were inactivated (Sinopharm‐BBIBP‐CorV, SinoVac‐CoronaVac) or the single shot vector vaccines (CanSinoBio) formulations. All COVID‐19 vaccines administered were found to reduce the risk of symptomatic disease.^20^ BBIBP‐CorV has been shown to reduce COVID‐19 severity.^21^, ^22^

There is limited information regarding the effectiveness of vaccinations administered in the context of SARS‐CoV‐2 variants in Pakistan also, factors associated with severe COVID‐19. Here we have investigated the risk factors, clinical presentations, and outcomes in relation to the VOC in patients admitted to our center with COVID‐19 between April 2021 and February 2022.

## 2 METHODS

This was a retrospective cross‐sectional cohort study of adults with a PCR confirmed COVID‐19 patients admitted to the AKUH (a 700‐bedded tertiary care hospital in Karachi, Pakistan), between April 2021 and February 2022. This study received approval by the Aga Khan University, Ethical Review Committee (study reference no. 2021‐6232‐19404).

All COVID‐19 cases had a SARS‐CoV‐2 PCR positive respiratory sample using the SARS‐CoV‐2 Cobas 6800 Roche assay, at the AKUH Clinical Laboratories, Karachi, Pakistan. The CT (crossing threshold) value of each positive SARS‐CoV‐2 sample was noted. The cases chosen for review were those for whom SARS‐CoV‐2 VOC testing had been conducted as part of a WHO supported genomic surveillance effort.^15^

Data were collected on symptoms at presentation, treatment, duration of illness, outcomes and COVID‐19 vaccinations. Laboratory and radiological data were collected for all cases. COVID‐19 severity was categorized into nonsevere in patients who have oxygen saturation ≥94% on room air, severe in those who have an oxygen saturation of ˂94% and require supplemental oxygen support and critical in those who have respiratory failure, shock and/or multiorgan dysfunction.^23^ WHO ordinal score for the severity of COVID‐19,^24^ length of hospital stay and in‐hospital mortality were also recorded. Individuals who had received both doses of a two‐dose vaccine regimen were categorized as “fully vaccinated,” those who had received one dose of a two‐dose vaccine regimen were categorized as “partially vaccinated” and those who had not yet received a single dose of COVID‐19 vaccination were classified as “unvaccinated.”

### 2.1 Identification of VOC

Isolates were screened for VOC; Alpha, Beta, Gamma, Delta, and Omicron lineage associated mutations using a PCR‐based approach.^15^

### 2.2 Statistical analysis

Median and interquartile range was reported for continuous variables such as age and length of stay and frequency and percentages were used to describe categorical variables such as gender and mortality. Comparison between categorical variables was determined using Chi‐square test or Fischer exact test where appropriate. All the tests were two‐sided and a *p* value of <0.05 was considered significant. Logistic regression analysis was performed to identify risk factors for the severity of illness and in‐hospital mortality and adjusted odds ratios (aOR) and their 95% confidence intervals (CI) were estimated. Initially, Univariate Logistic regression analysis was done to identify variables with a *p* ≤ 0.1, which were then analyzed in the Multivariable Logistic regression model. Data were analyzed using STATA™ version‐14.

## 3 RESULTS

### 3.1 Description of COVID‐19 cases

We studied the clinical characteristics and disease outcomes of 197 COVID‐19 patients admitted at AKUH between April 2021 and February 2022. Three COVID‐19 waves were observed in Pakistan during this study period (Figure S1A). COVID‐19 mortality was observed to be lower in the period between December 2021 and February 2022, as compared with the earlier waves between April and November 2021 (Figure S1B).

The median age of the patients was 55 years (interquartile range: 34–70 years) and 51.8% (*n* = 102) were males, Table S1. Hypertension (48%) and diabetes (41.3%) were the most common comorbid conditions. There were 23 pregnant patients (24.2% of females) and most of them were identified on screening before delivery. Amongst the pregnant women, 8 (34.7%) developed obstetric complications including intrauterine fetal demise in two cases (Table S2).

Upon admission, 48.2% of patients had nonsevere disease, while 51.8% had severe/critical disease. Fifty percent of patients had received systemic corticosteroids. Most patients survived (70.6%) till the time of discharge while 38 (19.3%) patients died and 20 (10.2%) left against medical advice (Table S1). Complications occurred in 27.4% of patients (Table S1). The most frequent complication was acute kidney injury in 14.7%, followed by myocardial infarction in 9.1% and pneumothorax/pneumomediastinum in 3.6% of patients.

Vaccination data was available for 154 (78.2%) and was missing for 43 (21.8%) patients. Available data showed that 92 (59.7%) study subjects were unvaccinated and 59 (38.3%) were fully vaccinated, while three individuals were partially vaccinated. Details of the administered vaccination were available in 49 cases; individuals had received inactivated vaccines (BBIBP‐CorV‐Sinopharm or SinoVac, *n* = 43), single shot vector vaccines (CanSinoBio, *n* = 3) or messenger RNA (mRNA) (Pfizer, *n* = 3) vaccinations. Hence, the majority (88%) of study subjects had received inactivated COVID‐19 vaccines.

The Omicron variant was found in 55.3% of COVID‐19 patients, with Alpha variants in 13.7%, Beta in 14.7% and Delta in 12.7% of cases. As these were in a relatively smaller number compared with Omicron, we combined cases with Alpha, Beta, Delta, and Gamma variants into a non‐Omicron group for further comparative analysis.

### 3.2 Factors associated with severe COVID‐19

We investigated the factors associated with severe COVID‐19 by stratifying the study group into those with nonsevere as compared with severe/critical disease. Age, clinical characteristics, vaccination status and infecting SARS‐CoV‐2 variants were all compared between the two sub‐groups. Most patients who developed severe disease were older than 50 years (*p* < 0.001), were male (*p* = 0.019), had a higher WHO ordinal score at admission or discharge (*p* < 0.001) and increased length of hospital stay (*p* < 0.001), Table 1. Vaccination (OR: 0.45; 95% CI: [0.23–0.88]), *p* < 0.001 showed an association with nonsevere disease, while infection with the Omicron variant was linked to nonsevere disease (OR: 0.42; 95% CI: [0.23–0.74]), *p* = 0.003.

**Table 1:** Clinical characteristics associated with nonsevere as compared with severe/critical COVID‐19.

Variables	Nonsevere (*n* = 95)	Severe/critical (*n* = 102)	Unadjusted OR (95% CI)	*p* value
Age range (years)				
<50	60 (63.2)	22 (21.6)	(Ref)	<0.001
≥50	35 (36.8)	80 (78.4)	6.23 (3.32–11.7)	
Median (IQR)	37 (28–60)	65 (50–74)	1.05 (1.03–1.07)	<0.001
**Gender**	***n* (%)**	***n* (%)**		
Male	41 (43.2)	61 (59.8)	(Ref)	0.019
Female	54 (56.8)	41 (40.2)	0.51 (0.30–0.90)	
Pregnant	20 (37.0)	3 (7.3)	0.13 (0.37–0.49)	0.001
Co‐morbid conditions				
DM	21 (22.1)	60 (58.8)	5.03 (2.69–9.40)	<0.001
HTN	30 (31.6)	64 (62.8)	3.65 (2.02–6.58)	<0.001
IHD	10 (10.5)	29 (28.4)	3.38 (1.54–7.39)	0.002
CKD	2 (2.1)	18 (17.7)	9.96 (2.24–44.2)	<0.001
**WHO ordinal score**	**Median (IQR)**	**Median (IQR)**		
At admission	3 (1–4)	5 (5–6)	8.02 (4.38–14.69)	<0.001
At discharge	1 (1–2)	3 (2–8)	7.52 (3.42–16.54)	<0.001
LOS	1 (1–3)	7 (3–15)	1.18 (1.10–1.27)	<0.001
**Outcome at discharge**	***n* (%)**	***n* (%)**		<0.001
Dead	1 (1.05)	37 (36.3)	70.1 (9.3–527.09)	
Alive	91 (95.8)	48 (47.0)	Ref	
LAMA	3 (3.2)	17 (16.7)		
**Vaccination status**	***n* (%)**	***n* (%)**		
Unvaccinated	38 (40.0)	54 (52.9)	Ref	
Fully vaccinated	36 (37.9)	23 (22.5)	0.45 (0.23–0.88)	0.019
*Unknown vaccination status* a	*21*	*25*		
**VoC**	***n* (%)**	***n* (%)**		
Non‐Omicron	32 (33.7)	56 (54.9)	Ref	
Omicron	63 (66.3)	46 (45.1)	0.42 (0.23–0.74)	0.003

*Note*: Non‐Omicron include Alpha, Beta, Delta, and Gamma variants.

Abbreviations: AKI, acute kidney injury; CI, confidence interval; CKD, chronic kidney disease; DM, diabetes mellitus; HTN, hypertension; IHD, ischemic heart disease; IQR, interquartile range; LAMA, left against medical advice; LOS, length of stay; MI, myocardial infarction; OR, odd ratio; VoC, variant of concern.

a Not included in multivariate analysis.

In a multivariate analysis, the independent risk factors for severe/critical disease were found to be age greater than or equal to 50 years (aOR: 5.73; 95% CI: [2.45–13.7]) and the presence of diabetes mellitus (aOR: 4.24; 95% CI: [1.82–9.85]), whereas being fully vaccinated (aOR: 0.25; 95% CI: [0.11–0.58]) were found to be protective against severe/critical disease (Table 2).

**Table 2:** Factors associated with COVID‐19 severity.

Severity of illness	Adjusted odds ratio	(95% Confidence interval)	*p* value
Age ≥50	5.73	(2.45–13.7)	<0.001
Presence of diabetes mellitus	4.24	(1.82–9.85)	0.001
Fully vaccinated	0.25	(0.11–0.58)	0.001

*Note*: A multivariable model was run to investigate factors associated with severity of illness using STATA software.

### 3.3 Factors associated with mortality

Factors associated with in‐hospital mortality were determined (after excluding 20 patients who left against medical advice). The median age of those who died was 64 years whilst those who survived was 48 years, *p* = 0.001, Table 3. Univariate analysis showed age greater than or equal to 50 years (OR = 5.73; 95% CI: 2.25–14.6), *p* = 0.001; the presence of co‐infections or secondary infections (OR: 2.45; 95% CI: 1.08–5.58), *p* = 0.032, presence of complications such as myocardial infarction (MI) (OR: 5.03; 95% CI: 1.69–14.9), *p* = 0.004, acute kidney injury (AKI) (OR: 3.78; 95% CI: 1.48–9.61), *p* = 0.005, having pneumothorax or pneumomediastinum (OR: 8.05; 95% CI:1.42–45.8), *p* = 0.019 and being unvaccinated (OR: 2.94; 95% CI: 1.02–8.39), *p* = 0.044, as factors associated with greater risk of death. Infection with Omicron as compared with non‐Omicron variants was associated with survival (OR: 0.28; 95% CI: 0.13–0.61), *p* = 0.001.

**Table 3:** Clinical characteristics of COVID‐19 patients associated with mortality.

Variables	Died (*n* = 38, %)	Alive (*n* = 139)	Unadjusted OR (95% CI)	*p* value	Multivariate analysis OR (95% CI)	*p* value
Median age (IQR) years	64 (55–75)	48 (30–69)	1.04 (1.02–1.06)	0.001		
<50	6 (15.8)	72 (51.8)	Ref			
≥50	32 (84.2)	67 (38.2)	5.73 (2.25–14.6)	<0.001	5.07 (1.92–13.42)	0.001
**Gender *n* (%)**						
Male	22 (57.9)	66 (47.5)	(Ref)			
Female	16 (42.1)	73 (52.5)	0.66 (0.32–1.36)	0.26		
**Co‐morbids *n* (%)**						
DM	20 (52.6)	49 (35.2)	2.04 (0.99–4.22)	0.054		
HTN	22 (57.9)	59 (42.5)	1.86 (0.90–3.86)	0.093		
IHD	10 (26.3)	22 (15.8)	1.90 (0.80–4.46)	0.14		
CKD	4 (10.5)	10 (7.2)	1.52 (0.45–5.14)	0.50		
**Co‐infection or secondary infections *n* (%)**	12 (31.6)	22 (15.8)	2.45 (1.08–5.58)	0.032		
**Complications *n* (%)**						
MI	8 (21.1)	7 (5.0)	5.03 (1.69–14.9)	0.004	5.11 (1.45–17.93)	0.001
AKI	10 (26.3)	12 (8.6)	3.78 (1.48–9.61)	0.005		
Pneumothorax/pneumomed	4 (10.5)	2 (1.2)	8.05 (1.42–45.8)	0.019		
Laboratory parameters						
At day 1						
CRP (mg/L) <100 (Ref)	17 (44.7)	59 (42.4)	Ref			
CRP ≥100	21 (55.3)	80 (57.6)	0.91 (0.44–1.87)	0.80		
D‐Dimer (mg/L) <1.5 (Ref)	7 (18.4)	40 (28.8)	Ref			
D‐Dimer >1.5	31 (81.6)	99 (71.2)	1.78 (0.72–4.39)	0.20		
**Vaccination status** a						
Fully vaccinated	5 (13.1)	47 (33.8)	Ref			
Unvaccinated	20 (52.6)	64 (46.0)	2.94 (1.02–8.39	0.044		
**VoC *n* (%)**						
Alpha	7 (18.4)	17 (12.2)	(Ref)			
Beta	8 (21.0)	18 (12.9)	1.07 (0.32–0.62)	0.90		
Gamma	3 (7.8)	3 (2.2)	2.42 (0.39–15.0)	0.64		
Delta	8 (21.0)	15 (10.8)	1.29 (0.38–4.43)	0.68		
Omicron	12 (31.6)	86 (61.9)	0.34 (0.12–0.98)	0.047		
**VoCs**	***n* (%)**	***n* (%)**				
Non‐Omicron	26 (68.4)	53 (38.1)	Ref			
Omicron	12 (31.6)	86 (61.9)	0.28 (0.13–0.61)	0.001	0.22 (0.10–0.53)	0.001

Abbreviations: AKI, acute kidney injury; CI, confidence interval; CKD, chronic kidney disease; CRP, C‐reactive protein; DM, diabetes mellitus; HTN, hypertension; IHD, ischemic heart disease; IQR, interquartile range; LOS, length of stay; MI, myocardial infarction; OR, odd ratio.

a A total of 20 patients who left against medical advice and 3 patients who were partially vaccinated were excluded.

A multivariate analysis run after adjusting for confounding and effect modification from other variables further confirmed in‐hospital mortality were found to be associated with age greater than 50 years (aOR: 5.07; 95% CI: [1.92–13.42]), *p* = 0.001, and the presence of MI (aOR: 5.11; 95% CI: [1.45–17.93]), *p* = 0.001. Infection with the Omicron variant was associated with survival (aOR: 0.22; 95% CI: 0.10–0.53), *p* = 0.001.

### 3.4 Viral loads were higher in Omicron infected individuals

**Figure 1:**
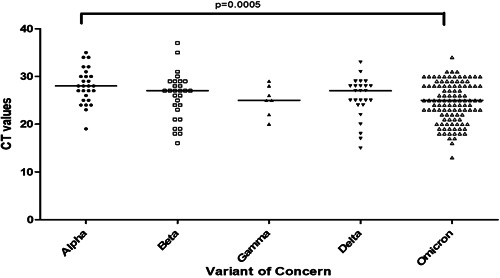
SARS‐CoV‐2 Omicron variants have lower CT values. The amplification thresholds (CT values) of the infecting VoC in SARS‐CoV‐2 positive nasal specimens of 197 patients was analysed according to the variant type. The graph depicts data for alpha (*n* = 27), beta (*n* = 29), gamma (*n* = 7), delta (*n* = 25) and omicron (*n* = 109) variants. Data shown is for the Orf1ab gene is depicted as per the SARS‐CoV‐2 Cobas 6800 Roche assay. The horizontal lines depict the median values for each group. The scatter plots depict interquartile ranges (10–90th percentile) for each group. MWU analysis, with 95% significance *p* < 0.05.

Higher SARS‐CoV‐2 viral loads have been associated with more severe disease. We determined the viral loads present in respiratory specimens and analyzed them according to the VOC. The CT of SARS‐CoV‐2 target gene amplification was used as a marker of viral loads. We found a significant difference between the CT values of the VOC, Figure 1, *p* = 0.015, Kruskal–Wallis analysis. In particular, the CT values of Omicron variants were lower, reflecting significantly higher viral loads as compared with the Alpha variant, *p* = 0.0005.

## 4 DISCUSSION

The correlation between COVID‐19 severity and SARS‐CoV‐2 variants has been a topic of significant discussion with limited information in the context of Pakistan. Our study was conducted through the pandemic waves in Pakistan characterized by ancestral followed by G clade, Alpha, and then Delta followed by Omicron variants.^14^, ^15^ We found a large proportion of patients were unvaccinated and nearly half the COVID‐19 admissions developed critical disease. Of those vaccinated, 88% had received inactivated virus vaccines. COVID‐19 patients infected with the Omicron variant exhibited milder symptoms upon presentation and had lower mortality rates compared to those infected with non‐Omicron variants. However, this protective effect was less pronounced in individuals over the age of 50. We found diabetes mellitus, hypertension, chronic kidney disease, age over 50 years, and critical disease on presentation were associated with higher mortality.

Our results regarding risk factors associated with severity fit with previous reports that showed comorbidities including diabetes mellitus, structural lung disease, cardiovascular disease, and hypertension were associated with an increased risk of severe COVID‐19.^25^

During the first year of the pandemic (2020) in Karachi, we had observed that severe to critical disease at presentation, and age more than 60 years and critical disease to be independently associated with mortality.^10^ This trend remained consistent in 2021 and 2022 except, that patients infected with Omicron variant had lesser odds of dying of COVID‐19 as compared with non‐Omicron variants, matching previous reports.^26^

One quarter of females with COVID‐19 were those pregnant. This high number of detected cases was due to the diagnostic screening practice at the AKUH which required testing of women who presened to the labor room or for elective surgical deliveries. The COVID‐19 severity in pregnant women was mild. However, it highlights the increased risk to mother and child in this cohort as has been shown by global studies on the impact of COVID‐19 on maternal health.^27^

We found vaccinated individuals to have a reduced risk of mortality from COVID‐19. Our data correlates with previous reports which have shown effectiveness of COVID‐19 vaccines in protection against severe disease.^19^, ^28^ It also fits with reports from Pakistan which showed BBIBP‐CorV vaccination was protective against severe COVID‐19 in Pakistan during the Delta wave.^22^ The relatively low rates of COVID‐19 vaccination we observed amongst patients could be due to delays in roll out of vaccinations or, hesitancy of individuals to be vaccinated.^29^

The majority of our vaccinated study subjects were infected with the Omicron variant likely due to the higher risk of breakthrough infections. Importantly, the Omicron variant had lower CT values associated with higher viral loads as compared with the Alpha variant. This differs from previous variants where genome‐wise analysis of SARS‐CoV‐2 have demonstrated higher viral loads to be associated with increased disease severity.^30^

A study from the United Kingdom also reported an increase in deaths due to Alpha variants.^31^ Studies from Brazil and Singapore had reported increased severity of disease in COVID‐19 patients with the Gamma variant and Delta variant, respectively.^32^ Reports show that Omicron variant caused less severe disease across multiple countries in the context of various COVID‐19 vaccinations.^13^ Reports from South Africa showed the Omicron wave to have reduced COVID‐19 related mortality in patients aged 50 years and above.^33^ In Europe, the CFR from infections with Omicron was significantly lower as compared with Delta.^34^

A limitation of our study is that it is from a single‐center tertiary care facility. Further, our sample size was relatively small, and we did not have complete vaccination details for all study subjects. Most patients were vaccinated with inactivated vaccines due to the local availibility COVID‐19 vaccinations. However, we provide valuable clinical evidence regarding the factors associated with disease severity and mortality, specifically in the context of VOC and protective effect of vaccinations. The protective effect observed aligns with the efficacy studies of different vaccines used in Pakistan, which have shown inactivated type of vaccinations were moderately effective against symptomatic COVID‐19.^20^, ^35^

Kandeel et al.^36^ from Egypt reported that patients infected with Omicron experienced less severe symptoms and a reduced likelihood of hospitalization. However, among those who were hospitalized, individuals with Omicron exhibited a more serious progression of the disease and higher mortality rates compared to patients hospitalized due to other variants.

The response to the emergence of Omicron variants was a strong recommendation to administer booster doses of vaccines.^37^ It is important to have effective vaccines targeting new SARS‐CoV‐2 variants, as they play a vital role in preserving lives and reducing hospital admissions.^38^ Many clinical trials have documented the utilization of inactivated or mRNA vaccines to combat the Omicron variant of SARS‐CoV‐2. The development of an Omicron‐specific COVID‐19 vaccine has already started, mostly by Chinese and US sponsors, and with clinical trials expected to end by 2023.^38^

Importantly, we show that for admitted patients, comorbid conditions, older age group and absence of vaccinations were all associated with increased risk of inpatient mortality. There was a high incidence of COVID‐19 in pregnant women in the population. It is evident that in Pakistan like other countries, COVID‐19 vaccination were key in reducing disease severity regardless of the SARS‐CoV‐2 variant involved. Further, our research shows that virus inactivated vaccines improved COVID‐19 outcomes. This is important in the context of vaccine availability as whilst new vaccines are being developed, these may not be readily available especially in low‐resource settings. Our highlights the significance of correlating clinical and epidemiological data with pathogen genomic analysis to provide insights for public health decision‐making.

## AUTHOR CONTRIBUTIONS

**Muhammad Zain Mushtaq**: formal analysis; investigation; writing—original draft. **Nosheen Nasir**: conceptualization; formal analysis; investigation; writing—review & editing. **Syed Faisal Mahmood**: formal analysis; investigation; writing—review & editing. **Sara Khan**: data curation; investigation; methodology; writing—original draft. **Akbar Kanji**: data curation; formal analysis. **Asghar Nasir**: data curation; formal analysis. **M. Asif Syed**: resources. **Uzma Bashir Aamir**: funding acquisition. **Zahra Hasan**: conceptualization; funding acquisition; investigation; project administration; writing—review & editing.

## CONFLICT OF INTEREST STATEMENT

The authors declare no conflicts of interest.

## ETHICS STATEMENT

This work complies with the policies for Ethical Consent as per Helsinki Declaration.

## TRANSPARENCY STATEMENT

The lead author Zahra Hasan affirms that this manuscript is an honest, accurate, and transparent account of the study being reported; that no important aspects of the study have been omitted; and that any discrepancies from the study as planned (and, if relevant, registered) have been explained.

## Supporting information

Supporting information.

Supporting information.

Supporting information.

## Data Availability

All data in the manuscript has been submitted in Supplementary files.
