# Supplementary material for: Exploring the relationship between SARS‐CoV‐2 variants, illness severity at presentation, in‐hospital mortality and COVID‐19 vaccination in a low middle‐income country: A retrospective cross‐sectional study
Source: Health Sci Rep. 2023 Dec 1;6(12):e1703. doi: 10.1002/hsr2.1703 (PMC10690835; doi:10.1002/hsr2.1703)
Supplement: Supplementary file 2 — Supporting information. [file HSR2-6-e1703-s002.pdf]

Supplementary Figure 1. Description of COVID-19 cases and deaths in Pakistan. The graphs depict data for COVID-19 between 1 April 2021 and 28 February 2022. A, Cases reported and B, Deaths reported. Source, John Hopkins, Corona Research Center https://coronavirus.jhu.edu/region/pakistan

A

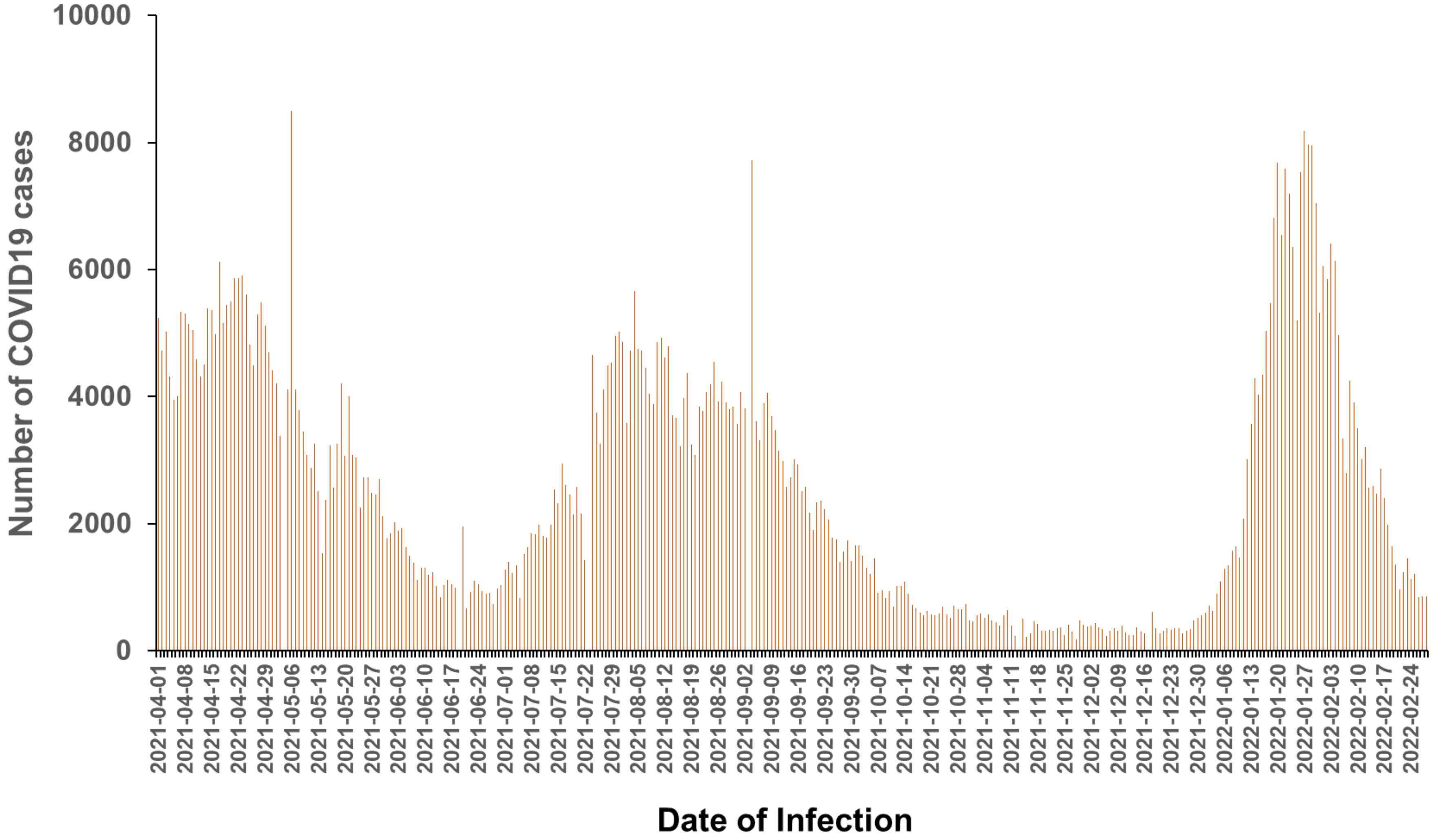

B

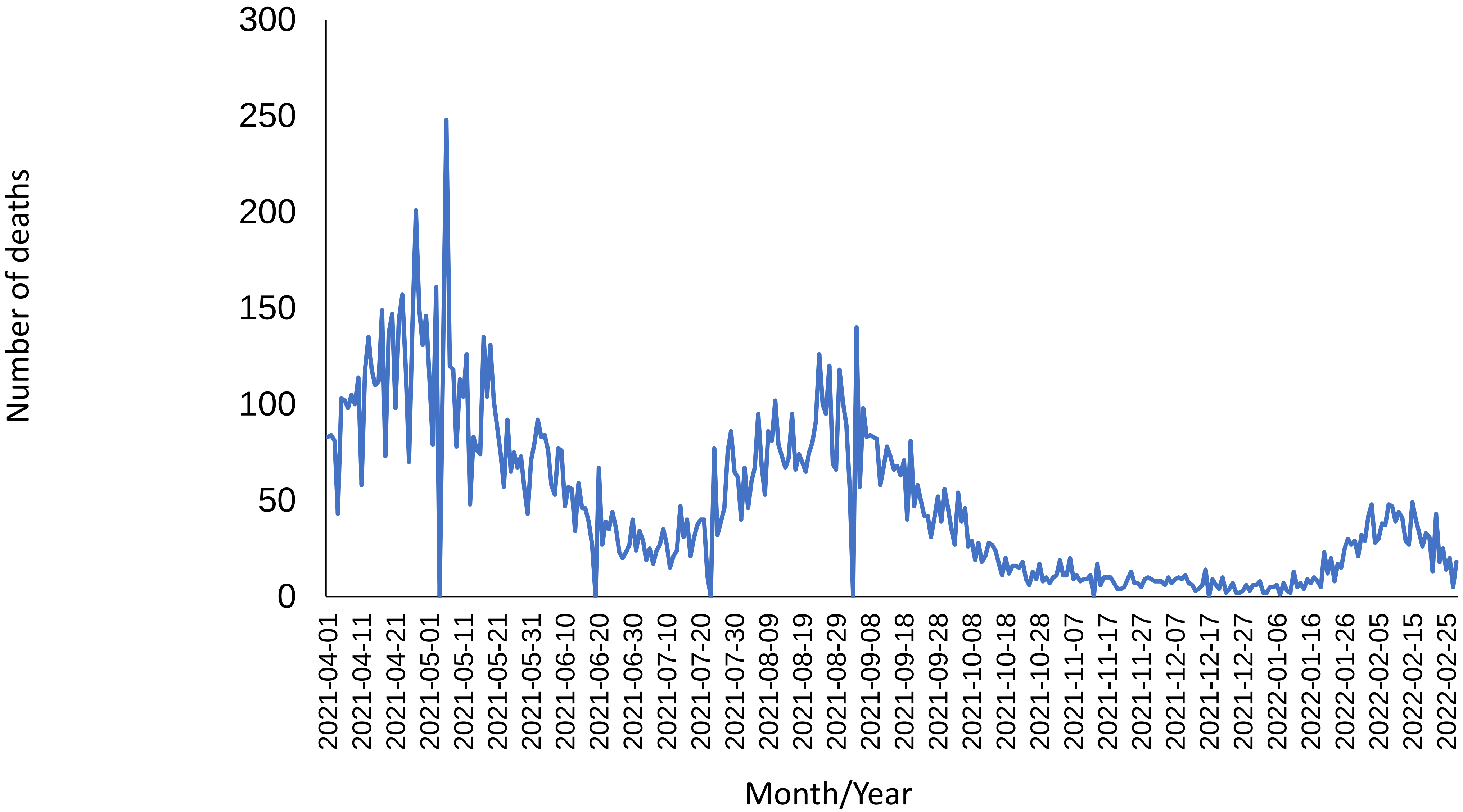

Number of deaths
300
250
200
150
100
50
0
2021-04-01
2021-04-11
2021-04-21
2021-05-01
2021-05-11
2021-05-21
2021-05-31
2021-06-10
2021-06-20
2021-06-30
2021-07-10
2021-07-20
2021-07-30
2021-08-09
2021-08-19
2021-08-29
2021-09-08
2021-09-18
2021-09-28
2021-10-08
2021-10-18
2021-10-28
2021-11-07
2021-11-17
2021-11-27
2021-12-07
2021-12-17
2021-12-27
2022-01-06
2022-01-16
2022-01-26
2022-02-05
2022-02-15
2022-02-25
Month/Year
